# Integrating the teaching role into one’s identity: a qualitative study of beginning undergraduate medical teachers

**DOI:** 10.1007/s10459-016-9694-5

**Published:** 2016-06-18

**Authors:** T. van Lankveld, J. Schoonenboom, R. A. Kusurkar, M. Volman, J. Beishuizen, G. Croiset

**Affiliations:** 1 Academic Centre for Human Behaviour and Movement, Faculty of Behavioural and Movement Sciences, LEARN!, VU University Amsterdam, De Boelelaan 1105, 1081 HV Amsterdam, The Netherlands; 2 VUmc School of Medical Sciences Amsterdam, LEARN!, VU University Medical Center, Amsterdam, The Netherlands; 3 Department of Education, University of Vienna, Vienna, Austria; 4 Research Institute of Child Development and Education, University of Amsterdam, Amsterdam, The Netherlands

**Keywords:** Dialogical self, Figured world, Identity, Medical teacher, Narrative

## Abstract

Beginning medical teachers often see themselves as doctors or researchers rather than as teachers. Using both figured worlds theory and dialogical self theory, this study explores how beginning teachers in the field of undergraduate medical education integrate the teacher role into their identity. A qualitative study was performed, involving 18 beginning medical teachers at a Dutch medical school. The teachers were interviewed twice and kept a logbook over a period of 7 months. The study shows that the integration of the teacher role into the teachers’ identity was hampered by the idea that teaching is perceived by others as a low status occupation. Some teachers experienced significant tension because of this, while others showed resilience in resisting the negative associations that were thought to exist regarding teaching. The teachers used five different identity narratives in order to integrate the teacher role into their identity, in which the positions of teacher and doctor or researcher were found to be combined, adopted or rejected in diverse ways. The five identity narratives were: (1) coalition between the I-position of teacher and other I-positions; (2) no integration of the I-position of teacher: holding on to other I-positions; (3) construction of the I-position of teacher and other I-positions as opposites; (4) coalition between the I-position of teacher and a third position of coordinator; and (5) meta-position: trivialising the importance of status. These identity narratives offer starting points for supporting undergraduate teachers during their early professional years.

## Introduction

Student-centred and problem-based education is intensive in terms of effort and staff. Unfortunately, continuity in the teaching workforce is limited as many staff members perform the teaching role only temporarily (Hirsh et al. 2007). This puts the quality of undergraduate education at risk, as developing expertise in teaching, especially in student-centred or problem-based education, requires several years of experience (Crosby 1996; Irby 2014). One of the explanations for the low continuity might be a low identification with the teaching role. Medical teachers often primarily identify with being a doctor or a researcher rather than with being a teacher (Bartle and Thistlethwaite 2014; O’Sullivan 2012). Insight into what explains this low identification however is limited.

Though there has been a growing interest in the identity formation of medical students and junior doctors in recent years (e.g. Barr et al. 2014; Dornan et al. 2015; Helmich et al. 2012; Monrouxe 2010; Vivekananda-Schmidt et al. 2015; Weaver et al. 2011), research on the identity formation of medical teachers is still scarce (Bleakley et al. 2011). This is a pity, since a smooth integration of the teaching role into their identity may not only encourage teachers to continue teaching, but also to invest in their development and teach with confidence (Monrouxe 2010; Wenger 1998).

### Theoretical framework

Identity involves an understanding of oneself in relation to others and one’s place in the world (Beauchamp and Thomas 2009). When people consider what to do or what to choose, they often implicitly consider this in relation to their understanding of who they are or would like to be. Identity therefore is an organising element in teachers’ lives used to legitimize and justify oneself (Beauchamp and Thomas 2009). In the current study, we adopt a combined socio-cultural and dialogical approach to the study of identity (Akkerman and Meijer 2011; Hermans and Kempen 1993; Holland and Lachicotte 2007; Penuel and Wertsch 1995). These approaches have four basic assumptions in common. First, identity is seen as a *construction* of the self. It is concerned with persuading others (and oneself) of who one is and what one values, and with positioning oneself in the world (Penuel and Wertsch 1995). For this positioning, people use discursive means like labels to categorise oneself or stories, and non-discursive means like wearing certain clothes. The second assumption is that identity is *social* in the sense that it is formed in relation to others: “identity formation employs a process of simultaneous reflection and observation, by which the individual judges himself in the light of what he perceives to be the way in which others judge him” (Erikson 1968, in Penuel and Wertsch 1995, p. 87). People construct their identities in relation to the communities they participate in; the membership of these communities constitutes their identity (Wenger 1998). The third assumption is that identity involves an *emotional* dimension (Meijers and Wardekker 2001; Penuel and Wertsch 1995). When teachers feel emotionally attached to their role, the category of ‘teacher’ becomes a more structural part of their thinking and reasoning, it becomes ‘part of who they are’ (Akkerman and Meijer 2011; Holland and Lachicotte 2007). The fourth assumption is that identity is *dialogical*. Both the socio-cultural and the dialogical approach acknowledge the existence of multiple identities, while at the same time expecting people to work at reducing the tensions between these different identities, or at least to be distressed when the identities conflict with each other, and to make an effort to synthesise and integrate these different identities into a coherent whole (Akkerman and Meijer 2011; Holland and Lachicotte 2007).

To understand how teachers integrate the teacher role into their identity and how they add it to the identity of doctor and/or researcher, we draw upon concepts from figured world theory, and dialogical self theory. Figured world theory (Holland et al. 1998; Holland and Lachicotte 2007) holds that identities are situated in socially produced, culturally constructed activities—what Holland et al. (1998) call *figured worlds*. Figured worlds are “socially and culturally constructed realms of interpretation in which particular characters and actors are recognized, significance is assigned to certain acts, and particular outcomes are valued over others” (Holland et al. 1998, p. 52). Before they begin teaching, beginning medical teachers’ identity is usually solidly grounded in the figured world of health care or research. Within these figured worlds, *collective stories* are told that form the cultural resources that beginning teachers draw upon to construct their identity. These beginning medical teachers also consider how the teacher role is perceived, recognised or valued by others in the figured worlds of health care, research or teaching (cf. Dornan et al. 2015). They ask themselves questions like: What does it mean to call oneself a teacher? How do others judge the teacher role? (Warin et al. 2006; Zittoun 2014). Several studies suggest that the collective stories in the figured worlds of health care and research do not position teachers and teaching in a positive way. Indeed, many medical teachers believe that teaching lacks prestige and recognition when compared to patient care and research (Hu et al. 2015; Huwendiek et al. 2010; Joyce et al. 2009; Kumar et al. 2011; Levinson and Rubenstein 2000; Sabel and Archer 2014). For beginning teachers, this might lead to difficulties in the process of integrating the teacher role into their identity. Such difficulties or tensions can lead to *identity* *dissonance*, which involves negative emotions such as having a low sense of self-worth or feeling frustrated (Monrouxe 2010; Warin et al. 2006).

Figured worlds theory provides insight into how and why tensions can arise in the integration of the teacher role into one’s identity. However, it does not help us to understand how new and existing roles can be integrated in such a way that these tensions are reduced. Therefore, we draw upon dialogical self theory (Hermans 2001, 2012, 2013; Hermans and Kempen 1993), which offers the conceptual tools necessary to understand this process. According to dialogical self theory (DST), identity formation can be seen as a negotiated process or as a synthesising activity between multiple positions (the so-called *I*-*positions*) that interact with each other in a dialogical fashion (Akkerman and Meijer 2011; Hermans 2012). In the case of beginning medical teachers, it is likely that they have adopted the I-position of doctor or researcher prior to the introduction of the new I-position of teacher, which hence engages in ‘dialogue’ with the I-position of doctor or researcher. This dialogue is not necessarily harmonious because, depending on the nature of the collective stories about teaching, there can be tensions between positions.

According to dialogical self theory, people develop *identity narratives*, discursive tools which can be used flexibly and creatively in order to integrate different identity positions into a coherent whole (Hermans and Hermans-Konopka 2010). Hermans and Hermans-Konopka (2010) describe three kinds of identity narratives. The first involves the creation of *coalitions* between two or more I-positions, for example by recognising that teaching adds to research or that research adds to teaching. Such coalitions between positions have the potential to generate strong motivation, since they take the interests of both positions into account and then combine them as parts of a more encompassing structure. The second kind of identity narrative concerns the creation of a *third position* that conciliates or mitigates the conflict between positions. In the case of medical teachers, this might involve adopting the position of ‘improver of the quality of patient-centred health care’, which lessens any potential conflict between the positions of teacher and doctor. The third identity narrative consists of taking a *meta*-*position*, which means distancing oneself and reflecting on the positions. These identity narratives can help in creating a dialogical space in which existing I-positions are negotiated, combined, or redefined, and new I-positions are created.

As discussed above, medical teachers’ identity can be expected to be solidly grounded in the figured world of health care or research before they start to teach. The question is how they add the I-position of teacher to the I-position of doctor and/or researcher, as well as what narratives they develop to overcome any possible tensions between the different I-positions. Thus far, no studies have described this process of integrating the teacher role into one’s identity. Further, the scarce empirical research on the identity formation of medical teachers that does exist is limited to experienced teachers (Hu et al. 2015; Kumar et al. 2011; Sabel and Archer 2014; Starr et al. 2003; Steinert and Macdonald 2015). These studies portray a dual picture. On the one hand, experienced teachers report feelings of marginalisation and a lack of self-esteem (Hu et al. 2015; Kumar et al. 2011; Sabel and Archer 2014; Stenfors-Hayes et al. 2010). They believe that in the field of academic medicine, teaching is perceived as a low-status occupation and that it is thus less valued than clinical work or research. The authors attribute these findings to the way academic careers are structured, as there is a strong emphasis on research productivity in academic medicine. As career entry in medical education is often unplanned and serendipitous, identification with the role of teacher often develops only gradually (Bartle and Thistlethwaite 2014; Hu et al. 2015). On the other hand, experienced teachers also generally see teaching as an important part of their identity (Hu et al. 2015; Kumar et al. 2011; Sabel and Archer 2014; Starr et al. 2003; Steinert and Macdonald 2015). Many see teaching as a responsibility or a moral commitment to give something back to the profession. Others mention a desire to contribute to the development of the next generation. The joy of teaching itself, that of watching students learn and taking pride in their accomplishments, is also mentioned, as are the opportunities that teaching provides for continuing one’s own learning.

The aforementioned studies primarily concern doctors who teach at the postgraduate level. Although they are primarily engaged in teaching, they also maintain some clinical practice and sometimes also conduct research. So far, there is only limited research available concerning teachers in undergraduate medical education. Due to the introduction of student-centred and problem-based curricula in undergraduate education, as well as the increased attention paid to clinical reasoning, communication skills, and professional behaviour, the academic workforce responsible for undergraduate teaching in recent years has not only grown strongly, but has also changed (Smith and Bunton 2012). In traditional undergraduate education, where large-scale lectures were given by professors, teaching was a highly regarded academic activity (Van Rossum 2012). However, in order to reach the number of tutors needed in student-centred and problem-based preclinical undergraduate education, much of the small group teaching is performed by junior staff, senior students, PhD students, postdoctoral researchers, or full-time teachers (Matthes et al. 2002). These positions do not necessarily embody the exclusivity and status that used to be ascribed to professors in the past.

The goal of the current study is to describe how beginning medical teachers in undergraduate education integrate the teacher role into their identity. Or, in terms of dialogical self theory, what identity narratives novice teachers use to integrate the I-position of teacher into their identity. Having insight into these identity narratives will provide medical schools with starting points for supporting undergraduate teachers during their early professional years.

## Method

We conducted an in-depth qualitative study from an interpretivist epistemological perspective, which assumes that meaning is constructed in the researcher-participant interaction, and which tends to rely on qualitative methods (Bunniss and Kelly 2010).

### Context

The study was conducted in 2012 and 2013 in a Dutch medical school. The school’s preclinical undergraduate programme in medicine consists of a three-year bachelor’s phase and a three-year master’s phase. It has an annual intake of 350 first-year students. Since 2005, the bachelor’s curriculum is a student-centred vertically integrated curriculum, with an emphasis on small group teaching. Small group teaching was introduced to promote active and self-directed learning, to foster the exploration of knowledge and to enhance students’ cooperative skills (Crosby 1996). The groups consist of 12 students and are led by teachers from all departments and from all levels of the hierarchy. The task of teaching is not only assigned to medical doctors, but also to PhD students and postdoctoral researchers. Increasingly, departments have taken to appointing full-time teachers to help meet their teaching requirements.

### Participants

Since we were exploring the identity narratives that are used by teachers who try to the integrate the teacher role into their identity, we sought a sample of beginning teachers that were actively exploring the identity of teacher. We therefore purposefully recruited participants from a voluntary university teaching qualification course (165 h, including modular courses and a workplace portfolio) and from teacher communities (i.e. groups of teachers who learn with and from each other during regular meetings in which they discuss any problems they face in their teaching) (Gercama et al. 2014; Van Lankveld et al. 2016). We only included teachers with no more than 5 years teaching experience (i.e. purposive sampling). Eighteen teachers participated in the study. Some had a medical background (MD degree), while others had a non-medical background (Masters in social sciences or health and life sciences). Eighty two percent were women, which is representative of the percentage of women attending the teaching qualification programme and the teacher communities (78 %), but not of the percentage of female teachers working in this medical school (52 %) (Table 1).

**Table 1:** Participants’ demographic information

	n	M (range)
Age^a^		30.8 (25–46)
Teaching experience^a^		1.3 (0–5)
*Gender*		
Male	3	
Female	15	
*Department*		
Basic sciences department (tasks: research and teaching)	8	
Clinical department (tasks: patient care, research and teaching)	2	
Teaching department (tasks: mainly teaching)	8	
*Time spent on teaching, research and patient care*		
100 % teaching	7	
50 % teaching, 50 % research, 0 % patient care	1	
10–30 % teaching, 70–90 % research, 0 % patient care	10	
*Teaching tasks (having more than one task is possible)*		
Teaching small groups (bachelor’s phase years 1–3)	18	
Bedside teaching (master’s phase year 1)	4	
Co-ordination or educational development	4	
Lecturing big groups	0	

^a^In years

### Data collection

We conducted two semi-structured interviews. In the first interview, we let the teachers reflect on their teacher role and on the role of teaching in their identity. We explored several areas: their teaching career so far, their professional identity, their stance towards their role as a teacher in relation to other roles, and their identity in relation to the environment (see “Appendix” for the interview guide). In the second interview, we further explored these issues by asking the teachers to elaborate on fragments from the weekly logbook that they had kept during the period between the interviews (7 months). The logbook fragments that were used as prompts in the second interview were selected by the researcher and concerned statements about how teaching was valued (see Table 2 for example questions). The interviews were conducted and audio recorded by TvL, an educationalist with experience using qualitative research methods. The average duration of the two interviews in sum is 137 min (range 93–198 min). The data collection and analysis occurred in an iterative process. After the first five interviews were completed, the data were analysed. This allowed early analytic insights to inform the subsequent data collection: these insights sensitized us to the identity narratives used by the teachers, which helped us to formulate better prompts in later interviews and make a sharper selection of logbook fragments later. After another 13 interviews had been conducted, we went back to the first 5 interviews for re-analysis.

**Table 2:** Examples of questions asked during the second interview

In your logbook, you wrote that you felt that in this medical school, “people tend to think that teaching is something we can all do, alongside other tasks”. Do you have this feeling often? What is it that makes you feel like this? What kind of things strengthen this feeling? What effect does it have on you? In your logbook, you wrote several times that you feel supported by your colleagues. Could you describe what it is that makes you feel supported? In your logbook, you wrote that you talked with a colleague who had applied for a clinician job and who had heard that she was “only teaching”. You wrote: “I regret that many doctors see teaching as an admission of weakness, as something for doctors who aren’t good enough”. Is it your impression that many clinicians see teaching as an admission of weakness? What kind of things strengthen this impression? What impact does it have on you?	

### Ethical approval

Ethical approval for this study was granted by the Ethical Review Board of the Dutch Association for Medical Education (NVMO-ERB reference number 169). The participants were informed about the scope and nature of the study, and they all gave written consent to participate. To maintain confidentiality, all of the data were transcribed and analysed anonymously by using pseudonyms.

### Data analysis

The data were transcribed by two student assistants and then checked for accuracy by TvL. The data analysis proceeded through several rounds of close reading and re-reading of the data, data reduction (by selecting, coding and summarising) and data display in within-case and cross-case matrices, and interpretation (Miles and Huberman 1984).

First, descriptive codes were assigned in Atlas.ti by TvL with the primary purpose of making the data accessible for later retrieval. The codes, such as *pride in teacher role*, *relation between teacher and doctor role*, *relation between teacher and researcher role*, and *ambitions for the future,* were developed inductively and then revised and refined by TvL. Descriptions of the codes and illustrative data extracts were discussed with JS. Ten interviews were also independently coded by JS. A discussion about the differences led to the further specification of code descriptions.

Next, segments were located in the original data that related to the participant’s identity. These were condensed and summarised in within-case matrices, one for each participant. The data reduction into within-case matrices helped us to select and focus on the data that related to identity and sort and organize it in a structured way (Miles and Huberman 1984). The data were summarized, while rich and representative original quotes were included. This resulted in a total of 44 pages of text.

Out of these matrices, we further summarised the data into one all-encompassing 15-page matrix that included all participants. The columns included: a narrative description of the participant’s identity, tensions experienced in integrating the I-position of teacher into one’s identity, and the identity narratives that were used to integrate the role of teacher into one’s identity. Representative quotes were included in the data (see Table 3 for an example of one row of the matrix). Displaying the data in a cross-case matrix allowed us to assemble organized data into an accessible, compressed form so that we could interpret the rich and representative quotes in the context of the complete picture of the participant (Miles and Huberman 1984).

**Table 3:** One of the rows of the all-encompassing matrix

General information	Identity profile	Tensions experienced	Identity narratives
Lucy 27-years-old, full-time teacher, master’s phase year 1	As a child, Lucy had always wanted to become a primary school teacher. But when she realised she was a good student, she thought that teacher education would not offer her enough of a challenge. That is why she enrolled in medical school instead, after some encouragement from her father, who had always wanted to be a doctor but who had never had the opportunity. During her studies, she taught. As part of her master’s programme, she did an elective internship in education, which is very uncommon, as such internships are not typically offered in the programme. There she felt completely at home (“This was the only time during my internships when I thought, ‘This is why I’m here. This is what I really want…. Finally, I can really be myself and don’t have to pretend all the time’”). Lucy would like to stay in education for the rest of her career. She has serious plans to pursue a PhD in medical education. In the long run, she would like to add an educational development focus to her current programme involving only teaching, and to become involved in curriculum development	Lucy feels that, especially with doctors, she is looked down on when she introduces herself as a teacher. According to Lucy, her decision to teach is not very well accepted. “When I’m with medical professionals, I find it important to mention that I’m a physician. Otherwise, they’re really quick to look down on you. They treat you as though you have no ambition. They’ll ask me, ‘Do you teach because you want to be a specialist?’ When I answer, ‘No, I teach because it’s what I *want* to do,’ there’s always that awkward silence that follows. I find it very uncomfortable”	When Lucy introduces herself, she says (half jokingly, half seriously) that she wants to become an educational director at a university. She does this in order to prevent awkward silences and to clarify that she does have ambition, as well as to confirm that she takes teaching seriously

The final data analysis entailed a combination of micro- and macro-analysis of the rich and representative quotes that had been included in the within- and cross-case matrices. (Akkerman and Meijer 2011; Monrouxe 2010), in order to uncover and combine two layers: (1) the identity narratives that teachers used to maintain a coherent identity; and (2) the collective stories from which they were drawn and the way these were embedded in the figured worlds of teaching, research and health care. In our case, the micro-analysis entailed an analysis of the narratives teachers adopted to integrate the I-position of teacher in their identity, and how they adopted and shifted between the I-positions of teacher, researcher and doctor (Akkerman and Meijer 2011). The macro-analysis on the other hand, entailed an analysis of the collective stories being told about teaching in the figured worlds of research, teaching and health care, as echoed in the participants’ accounts. By combining the two, an understanding was reached regarding how the collective stories impinged on the teachers’ identity. The outcomes were discussed with other researchers, first with JS and later with two researchers from outside the research team, who conducted a peer review of the analysis of the cross-case matrix. One was an expert in qualitative methods and one was an expert in studying identity from a dialogical self theory perspective. Both researchers were independent from our research team and therefore held a critical distance to the data. Interim interpretations of the analysis were jointly discussed by all members of the team.

To enhance the study’s credibility and trustworthiness, multiple researchers were involved in the analysis, both from within and from outside the research team (triangulation of researchers) (Malterud 2001). In order to prevent ourselves from imposing our own values and drawing conclusions too early, we repeatedly read the data and checked back in the original data several times. We also deliberately searched for disconfirming evidence and paid extra attention to outliers, extreme cases, and surprises in order to prevent a ‘premature closure effect’ (Robson 2002). This led to more nuanced and refined explanations. The results of the study were discussed by the research team several times, allowing for the analyses to be challenged and contested. The research team consisted of researchers with backgrounds in both medical education (GC, RK) and general education (TvL, JS, MV, JB). The diverse backgrounds of the members of the team helped us to be somewhat removed from the medical education context, while at the same time allowing us to be sufficiently informed to understand the details of the participants’ context.

## Results

During the initial data analysis, it became clear that half of the teachers experienced tensions in the process of integrating the teacher role into their identity. These tensions will be described in the first section, since they seem to form a background against which many of the identity narratives that the participants used should be interpreted. We will then describe these identity narratives in the second section.

### Tensions between the I-positions of teacher and doctor or researcher

Several of the undergraduate teachers experienced tensions in relation to their perception that teaching is regarded as inferior to the two other roles, namely clinical work and research. These tensions were experienced by two different groups of undergraduate teachers.

The teachers with a master’s degree in medicine, on the one hand, held the notion that others in their environment regarded teaching as inferior compared to *clinical work*. They often had to explain why they choose teach, especially to others with a medical degree. Conversations about their decision to become a teacher were marked by uncomfortable silences or subtle remarks suggesting that teaching is regarded as “something for failed doctors”:

When I say that I’m a teacher, people with a medical background sometimes respond a bit like…. How should I put this politely? As if I had made an inferior choice, a B-choice. They make you feel like they’re thinking, ‘Oh, so you work in education instead of in a hospital? Why is that? Were you unable to cope in a hospital?’ They don’t literally come out and say that, but that’s the attitude they radiate. (Lotte, 25, full-time teacher, MD degree)

In particular, those teachers who had finished their postgraduate education and done clinical work for several years before making a career change to full-time teaching struggled with feelings of guilt and shame. Will, for example, does not tell other clinicians that her career change to teaching is definitive, implying that it is shameful to be a teacher. Will addresses that she does not see herself as a loser (implying that, in a way, she does), but that she does feel guilty that she teaches:

When I’m talking to outsiders, I always say, ‘Yes, I stopped working as a doctor for my child’s sake, and I’ll go back when my child is older.’ But *no way* … I don’t want to go back *ever* again. But I don’t want outsiders to know that yet.

*Why not?*

Well, because deep down, I do feel like you’re a doctor for life. I feel sort of … well, it’s not that I see myself as a loser, but I do feel guilty sometimes about all those other doctors. […] I find myself thinking, ‘I gave up and left it up to others to do what I’m supposed to be doing.’ […] You become a doctor for life, and here I am doing nothing with my MD. (Will, 41, full-time teacher, MD degree)

Teachers from the basic sciences departments who had non-medical backgrounds (psychologists, biomedical scientists, neurological scientists, anatomists), on the other hand, wrestled with the collective story that *research* is seen as more important than teaching. They reported that colleagues from their departments regarded research as the central task, and that such colleagues see teaching as “a burden” that distracts them from their “actual” work. Some of the teachers showed much frustration because of this, marked by a strong intonation and repetition of arguments. They stressed that reward systems in the higher education context are focused on publications, which they interpreted as an implicit message that research counts and that teaching does not. Especially the teachers working in departments with a strong research culture experienced strong tensions, such as frustration, loneliness, and a low sense of self-worth. They report that in their departments, colleagues (including professors) regard teaching as unimportant:

I feel as though teaching is not really appreciated. They simply don’t appear to understand the importance of good teaching…. It seems as though the only goal is to get those hours filled in [in the schedule] …. It doesn’t matter who does the job, how it’s done, or what gets done… […] It’s as though they think that, because I like teaching, that I’m pursuing some sort of hobby, which shouldn’t be allowed to take up too much time. (Lieke, 34, post doc, health/life sciences degree)

To sum up, the beginning undergraduate teachers were confronted with two collective stories about teaching, both of which involved rather negative connotations of the role of teacher. They experienced that within the figured world of health care, a collective story is told about “teaching being for failed doctors who cannot meet the standards of clinical work”, whereas within the figured world of research, a story is told that “teaching does not count as much as research”. Within both figured worlds, the role of teacher is not explicitly valued. These collective stories made it hard for the teachers to adopt the I-position of teacher. This led to conflicts for half of the teachers, not only between the I-position of teacher and the collective story about teaching, but also between I-positions. Although almost all participants acknowledged the collective stories described above, some experienced stronger tensions than others. To integrate the conflicting identity positions into a coherent whole, the teachers used a variety of identity narratives, which are described in the next section.

### Identity narratives used to integrate the teacher role into one’s identity

**Fig. 1:**
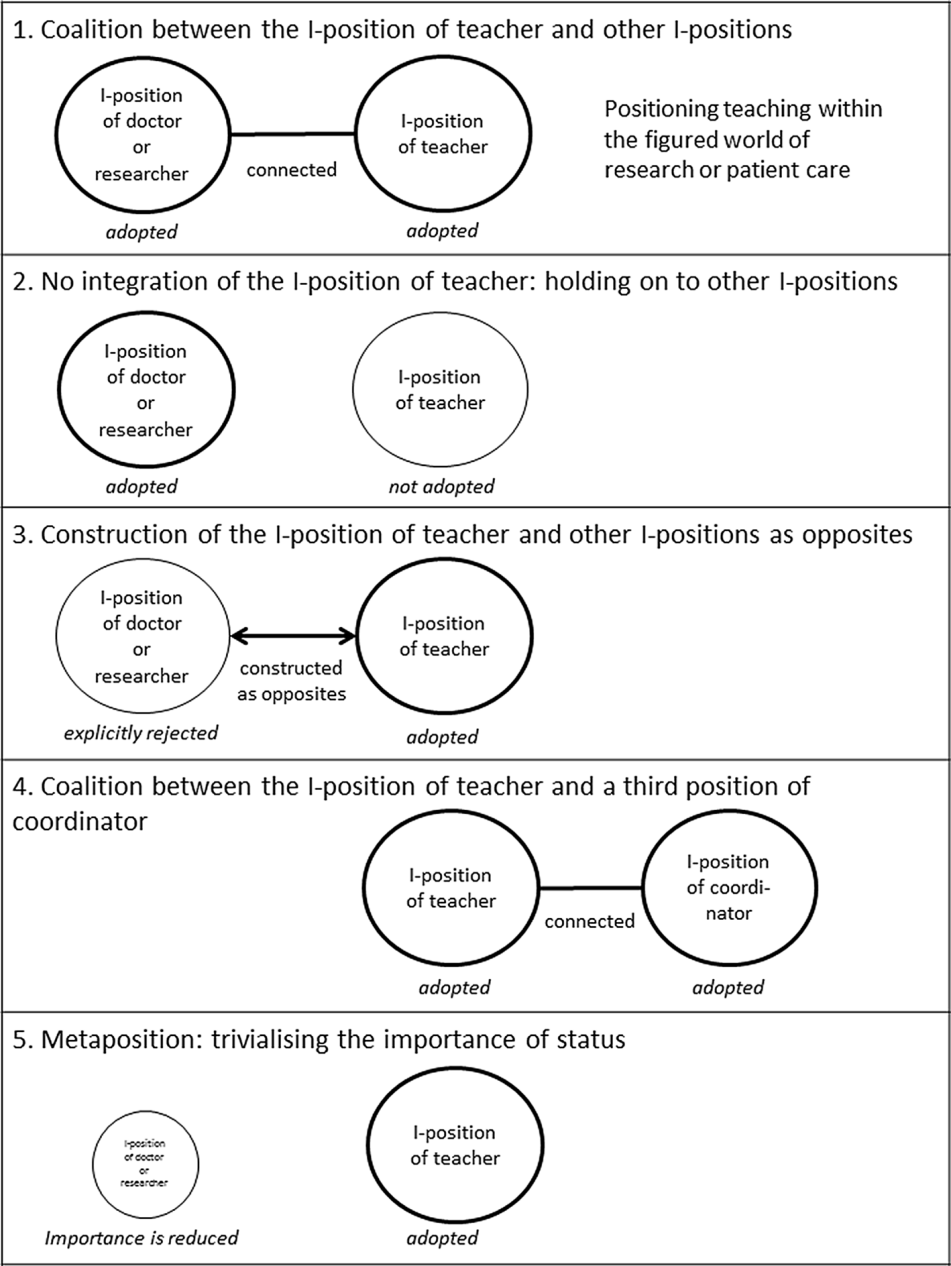
Identity narratives used to integrate the I-position of teacher into one’s identity

We found five identity narratives that beginning undergraduate teachers used to integrate the teacher role into their identity: (1) coalition between the I-position of teacher and other I-positions; (2) no integration of the I-position of teacher: holding on to other I-positions; (3) construction of the I-position of teacher and other I-positions as opposites; (4) coalition between the I-position of teacher and a third position of coordinator; and (5) meta-position: trivialising the importance of status. A schematic representation of these narratives is given in Fig. 1.

### Narrative 1: coalition between the I-position of teacher and other I-positions

The first identity narrative encompassed a *coalition* (Hermans and Hermans-Konopka 2010) between the I-position of teacher and that of doctor or researcher. Eight teachers used this narrative, in which it is argued that teaching helps one to achieve one’s ambitions as a doctor or researcher.

Some teachers emphasised the similarities between teaching and patient care. They argued that through developing teaching skills, they will become better doctors. In other words, the decision to teach was justified by emphasising its value to the figured world of health care in which the doctor I-position is central:

I think that teaching will ultimately help me to become a better doctor, because it helps me learn how to explain things in very basic terms that people can understand. And that’s a useful skill for interacting with patients. (Margot, 24, PhD student, MD degree)

Other teachers emphasised that being engaged in education is a good way to build a career in academia or else they argued that teaching is a way to introduce their research topic to clinical practice. Teaching was thus related to their commitment to research. For example, one of the teachers from a basic sciences department, not being a doctor herself, argued that she is proud of making a contribution to the medical world by teaching about doctor–patient interaction on the undergraduate programme:

I’m very proud of the connection between my own field of research and clinical practice. It’s nice to be able to use that knowledge in professional training, even though it’s quite theoretical. Quite often, I hear doctors say that they never received that kind of teaching during their own medical training, and that they consider it important that we’re offering it to the current generation of students. (Frances, 32, post doc, social sciences degree)

In this first narrative, the I-position of teacher is built on the I-position of doctor or researcher, and it serves the ultimate goal of strengthening the latter. Teaching is hence given a place within the figured world of research or health care.

### Narrative 2: no integration of the I-position of teacher: holding on to other I-positions

Six of the teachers held on to their primary I-position as doctor or researcher and did not adopt the I-position of teacher. Some of them are PhD students who see teaching as a temporary task. Thus, for them, the I-position of researcher remains primary. Others, however, are full-time teachers with a degree in medicine, who still, even though they work full-time as a teacher and do not practice as a doctor any longer, hold on to their I-position as a doctor. These teachers primarily consider themselves to be doctors:

I introduce myself as a doctor/teacher. I like to put it that way because I really want to hold on to my role as a doctor. I often say ‘I’m a [specialist], but I’m currently tasked with teaching junior interns in the hospital.’ And whenever I’m standing at a bedside [as a teacher on the first year of the master’s programme] to teach students how to examine patients, I do feel that I’m a doctor. At such moments, I don’t really feel like a teacher. (Suzanne, 46, full-time teacher, MD degree)

In this narrative, the I-position of teacher is not integrated into the teachers’ identity. Instead, it is omitted.

### Narrative 3: construction of the I-position of teacher and other I-positions as opposites

Four of the teachers used a narrative in which they distanced themselves from the figured world of health care or research and rejected the I-position of doctor or researcher. The teachers from the basic sciences departments, for example, made critical remarks about the competitiveness of academia and the high demands that they face. Likewise, the teachers with an MD degree criticised the hierarchical culture in the hospital and the long working hours:

[At the hospital] They simply disagree with your request, and ultimately, they want to hear it from your supervisor…. The hierarchy problem gets to me sometimes. […] Compared to the hospital, I think teaching really is a warm bath. (Lotte, 25, full-time teacher, MD degree)

In this narrative, the boundaries between the different I-positions were sharpened, since the I-position of teacher on the one hand and the l-position of doctor or researcher on the other were either implicitly or explicitly constructed as opposites.

### Narrative 4: coalition between the I-position of teacher and a third position of coordinator

In the fourth narrative, which was used by three teachers, coordination and educational development responsibilities were emphasised. The teachers who used this narrative adopted the I-position of teacher without necessarily rejecting the I-position of doctor or researcher. By stressing their co-ordinating responsibilities, they instead adopted a *third position* of co-ordinator, which then gives status to the I-position of teacher. They positioned themselves in the figured world of teaching, in which these responsibilities seem to be considered valuable. Teachers with coordinating responsibilities saw these tasks as a step forward in their career and as a way to be recognised by others:

I think that once I get more responsibility to develop the course, people will see me more as the one to turn to for assistance. I’m already noticing that the people in charge of semester co-ordination are now starting to recognise me. (Margriet, 35, full-time teacher, MD degree)

One teacher who used this narrative is Lucy, a full-time teacher with a master’s degree in medicine. Lucy aspires to a career in education, but feels that this choice is looked down on by others (“They treat you as though you have no ambition”). To prevent uncomfortable silences, she now (half jokingly, half seriously) says she wants to become the educational director of the medical school. By using narrative four as a future perspective, without actually having taken on those tasks yet, Lucy found a creative way to counterbalance the negative collective story that “teaching is for failed doctors”.

### Narrative 5: meta-position: trivialising the importance of status

Finally, four of the teachers adopted the identity narrative of questioning or trivialising the role of status altogether. In this fifth identity narrative, the teachers adopt a *meta*-*position* (Hermans and Hermans-Konopka 2010) by taking a certain distance from the collective stories within the figured world of research or health care. By adopting this meta-position, the teachers render ‘status’ less important, thereby making it easier to integrate the I-position of teacher into their identity. Will, for example, distances herself from people who think doctors are important, thereby putting the higher status of patient care and research into perspective:

No-o, I never say what kind of work I do. I notice that if I say I’m a doctor, people treat me differently, and I really hate that. Or they are shocked, because they hadn’t expected that I’m that highly educated. I notice that people sometimes… well how to put this… get a bit more friendly. And I don’t like that. I believe we should accept people the way they are and avoid judging them based on their education, ethnic background or anything else. I am not the kind of person who thinks that work is the most important thing in life. (Will, 41, full-time teacher, MD degree)

## Discussion

This study aimed to explore how beginning teachers in undergraduate medical education, who often see themselves as doctors or researchers rather than as teachers, integrate the teacher role into their identity. The analysis revealed that the smooth integration of the teacher role into identity is hampered by the negative messages concerning the value of their educational undertakings that teachers encounter in the figured worlds of health care and research. Half of the teachers experience strong tensions due to these negative collective stories, but the other half show agency to resist, transform or adapt them. In order to deal with these stories, the teachers used five identity narratives. Three of the narratives concerned the identity narratives as described by Hermans and Hermans-Konopka (2010): coalitions, third positions, and meta-positions. We also found two additional identity narratives: one in which the different I-positions were constructed as opposites and one in which the I-position of teacher was not at all integrated into the teachers’ identity.

In recent decades, many medical schools have transformed their preclinical undergraduate programmes into vertically-integrated, problem-based or student-centred curricula (Bandiera et al. 2013; Elliott 1999). Due to this, more teachers are needed to facilitate small group learning. As a consequence, a significant proportion of teaching is undertaken by young researchers who teach as only a small part of their job, or by former doctors who have stopped doing clinical work and now teach full-time (Matthes et al. 2002). This transition seems to have had consequences for the prestige of teaching: it is no longer seen as a highly regarded academic activity, as was the case when teaching was mainly performed by professors (Van Rossum 2012). Instead, the teachers in our study experience that in the figured worlds of health care and research, collective stories exist that exhibit rather negative connotations to teaching. These collective stories hamper the smooth integration of the teacher role into one’s identity.

Half of the novice medical teachers experienced strong *identity dissonance* due to the negative collective stories. They experienced disharmonious and negative emotions like frustration, guilt and a low sense of self-worth. The identity dissonance is problematic, since it might hinder the teachers to practise with confidence (Monrouxe 2010). On the other hand, the study also showed that several teachers managed to resist the negative associations surrounding teaching and instead created their own identity story. These teachers showed *resilience*; they resisted, transformed or adapted the existing stories. This resonates with the notion of *agency*, the capacity of individuals to shape their identity and remake cultural practices in transformative ways (Billett 2006; Eteläpelto et al. 2013).

The teachers positioned themselves in relation to three figured worlds: that of teaching, research and health care. Remarkably, the figured world of teaching was far less prominent than the other two. It apparently did not seem to offer many appropriate resources to identify with for beginning undergraduate teachers. Compared to postgraduate teachers described in other studies, the undergraduate teachers in our study seemed to have fewer positive stories available to identify with (Kumar et al. 2011; Steinert and Macdonald 2015). One reason for this could be that stories like ‘giving something back to the profession’, ‘contributing to the next generation’ or ‘it’s a joy to see students learn and grow’ (Steinert and Macdonald 2015) do not apply to novice undergraduate teachers. For the younger participants in our sample, for example, it might be too early to use these stories, since many were in between their master’s and postgraduate training, thus being the next generation themselves. They perhaps felt too much of a beginner to see teaching as an undiluted pleasure.

Based on our study, it seems that the figured world of teaching is a less pronounced and less developed figured world than the figured worlds of research and health care. This is in line with recent studies, in which the figured world of teaching has been described as an indistinct practice which lacks visibility, since rewards for teaching are largely intangible and symbolic and career pathways in medical education are unclear (Hu et al. 2015; Sabel and Archer 2014). Moreover, the figured world of teaching lacks a well-defined scope of activities. The word “medical education” for example, is interpreted in different ways: some see it as synonym for pursuing teaching activities, whereas others see it as synonym for pursuing educational research (Hu et al. 2015; Sabel and Archer 2014). This ambiguous and not very visible character of the figured world of teaching might hinder medical teachers in identifying with the teaching role.

In this study, we found five different identity narratives being used to integrate the teacher role into one’s identity. According to Hermans and Hermans-Konopka (2010), the first identity narrative of creating coalitions between positions has the most potential in the long run, in creating enduring motivation. In such a coalition, the interest of the role of teacher is taken into account, as well as the interest of the role of researcher or doctor. Creating a hybrid coalition between these two positions therefore allows the teachers to fall back on both figured worlds and to use both I-positions strategically and flexibly.

Our study displayed that identity formation is a dynamic, dialogical, and emotional process of positioning and negotiation. We found it to be dynamic, since the teachers did not simply repeat or adopt the collective stories they were faced with, but also took an active stance against them or creatively developed their own answers. We found it to be dialogical, as teachers adopted the I-positions of teacher, researcher and doctor in a dialogical (and not harmonious) way. Finally, we found it to be emotional, due to the strong tensions that half of the teachers experienced—though this appeared to be the case for only half of the teachers.

### Strengths and limitations

One strength of this study is that it is, to the best of our knowledge, the first to address the consequences of problem-based or student-centred curricula in relation to the identity formation of the teaching workforce. It is also one of the first studies that explores the ‘hidden curriculum’ for faculty: the subtle and sometimes indirect ways in which teachers come to understand what it means to be a teacher in academic medicine. This hidden curriculum is neither well-documented nor well-understood (Hafler et al. 2011). A theoretical contribution of this study is that it shows that the combination of figured worlds theory and dialogical self theory can make visible how the cultural and the personal are interrelated. It shows how the presence of negative collective stories or the lack of positive collective stories can lead to tensions experienced at the personal level. Furthermore, the study shows the dynamic nature of identity formation, which is sometimes hindered by negative collective stories, although at the same time individual teachers have agency to reshape or resist these stories and offer their own answer to them.

It is important to note here that in this study beginning medical teachers were included who voluntarily participated in a university teaching qualification course or in a teacher community of tutors discussing teaching experiences. These groups were likely to be actively exploring the I-position of teacher. We purposefully selected this group, since we were interested in the question what identity narratives this particular group of teachers used. It is important to note though, that we did not aim to generalise across the complete group of beginning medical teachers. It is likely that teachers who do not aspire to a career in teaching, use other identity narratives than the participants in our sample did. Further research would be needed to find this out, as well as what identity narratives are used by experienced, well-established medical teachers.

We also would like to acknowledge here that our study is strongly bound to the Dutch context, where careers are mainly advanced based on research, and where the possibilities for promotion based on pedagogical merits or contributions to educational innovation are still limited (Ten Cate 2007). Maybe in the Netherlands, more than in other countries, the undergraduate teaching workforce consists of a relatively large amount of teachers who are not clinically active. Specifically, it is quite common in the Netherlands for young MDs to teach and/or do research before residency (Capaciteitsorgaan 2015). This particular group of young MDs might only be tentatively exploring a teacher identity, before they start their career in patient care.

Additionally, the medical school where this study was performed was still in the transition phase from a traditional to a student-centred curriculum. Although the new curriculum was introduced 7 years before the study took place, the recognition of teaching had not (yet) changed significantly, neither formally in terms of promotion and tenure, nor informally in terms of status.

Furthermore, we like to point out that the findings might have been influenced by the fact that there was an overrepresentation of women in our sample. Earlier research has shown that while male and female junior doctors have similar attitudes toward undergraduate teaching, women feel less confident than men (Prichard et al. 2011). This might have coloured the findings, although we do not know whether women and men integrate the teacher role into their identity differently. Further research among male teachers would hence be valuable.

Finally, we would like to make some reflective comments here on the impact of the researchers on the study. A researcher’s background affects both the researcher-researched relationship and the findings that are considered most appropriate (Berger 2015). As the interviewer in this study is an educationalist, she represented the figured world of teaching. This might have impacted the way the participants presented themselves. However, we believe that the educational background of the interviewer could also be seen as a strength, since it helped in achieving rapport with the participants and in approaching the topic from a fresh angle. In order to ensure we would comprehend the specific context of medical education (not being familiar with the culture of academic medicine), we included two medical education experts in the research team.

### Implications for practice and future research

What can medical schools do to support novice medical teachers? We argue that the smooth integration of the teacher role into teachers’ identity can be enhanced by actively developing a figured world of teaching. This can be reached in four ways.

First, a figured world of teaching can be developed by actively introducing or reinforcing positive stories about teaching, since these are the resources for beginning teachers to identify with. It is important for medical schools to create opportunities for beginning teachers where they can tell their stories to others and hear stories from positive role models. Faculty development could play a role in this. From the field of Finnish teacher education, positive experiences are reported with identity coaching programmes that help individuals to adopt new identity positions (Kalliola and Mahlakaarto 2011). These positive experiences might be valuable for medical education.

A figured world of teaching can also be developed by creating traditions, events and activities (Holland et al. 1998). In order for the figured world of teaching to become more visible, it seems important that activities and events are introduced, in which teachers have a central role, and in which the significance of their role is celebrated. Ceremonies in which teaching qualification diplomas are granted could for example play such role, as well as festive ceremonies in which teaching awards and distinctions are conferred. Such events could provide beginning teachers a platform where they can find role models to identify with.

An additional way to develop a figured world of teaching is to build community. In a community, teachers find a sense of connectedness with others and a sense of recognition for their competences, which makes them feel appreciated (Van Lankveld et al. forthcoming; Wenger 1998). A community also provides a platform for sharing stories. Staff development activities can provide such community for beginning medical teachers, though this might not necessarily lead to an enduring community. We suggest to facilitate enduring contacts between teachers, like in associations for medical teachers (Cooke et al. 2003; Dewey et al. 2005),

Finally, in order to truly build a figured world of teaching, it is important to create reward systems and promotion structures in which teachers feel valued. Despite the repeated recommendation to reward teaching (Christakis 1995), positive experiences are still scarce.

For future research, it would be interesting to explore whether initiatives which help to build a figured world of teaching have a positive effect on the identity formation of teachers. Initiatives have recently been taken in this direction, like the introduction of a teaching portfolio for tenure and promotion, teaching fellowships (Engbers et al. 2015; Lown et al. 2009; Steinert et al. 2010), grants for educational innovation, and educational research (Adler et al. 2015). It would be interesting to investigate whether these initiatives change the collective stories about teaching and thus make better and more suitable identity narratives available.

A further suggestion for future research would be to explore the situated practices of the department, in which the collective stories about teaching are maintained or changed, since it is at the departmental level where the collective stories are re-told and redefined. Ethnographic studies seem especially suited to this kind of research, since they allow the investigation of the situated practices of departmental culture (Hammersley and Atkinson 2007).

## Appendix 1: Interview guide: interview 1

*Teaching career so far*

Can you please describe your teaching career so far?

- What tasks?

Why did you decide to go into teaching?

If you were to draw a line representing your confidence over the course of your teaching career, what would it look like?

- Can you elaborate on the periods you felt less confident? What factors contributed?
- Can you elaborate on the periods when you were more confident? What factors contributed to your increased confidence?

*Professional identity*

How do you introduce yourself to others?

- As a teacher, a researcher or a doctor?
- Why?

What kind of teacher are you?

- What kind of teacher would you like to be?
- Do you think there are different kinds of teachers? If so, what kinds?

How do you think others see teachers?

Do you ever feel proud to be a teacher?

- What makes you feel proud? What factors contribute to this feeling?

Who are role models in your work?

- Who are role models in your work as a teacher?
- What is it you appreciate about them?

What would you be most proud of, if you would have achieved it in 5 or 10 years?

*Stance towards their role as a teacher in relation to their other roles*

If you had to grade how much you like the different tasks you do (teaching, research, patient care), what grades would you give? Why?

If you were able to choose between receiving a prize as teacher or as a doctor or as a researcher, which prize would you value the most? Why?

*Identity in relation to the environment*

Do you believe that you can realise your ideals regarding teaching?

- Do you ever feel hindered by your environment? What happens in those cases? What would you like your environment to do?
- Do you ever feel supported by your environment? What happens in those cases?

What tips would you give to younger colleagues who would like to build a career as a teacher?

- What would you suggest them to do?
